# (R)evolution: toward a new paradigm of policy and patient advocacy for expanded access to experimental treatments

**DOI:** 10.1186/s12916-016-0586-6

**Published:** 2016-02-29

**Authors:** Melissa Hogan

**Affiliations:** Saving Case & Friends, Inc, P.O. Box 384, Thompson’s Station, TN 37179-7900 USA

**Keywords:** Expanded access, Compassionate use, Patient advocacy, Social media, Experimental treatment, Clinical trial, Right-to-try, Terminal illness, Rare disease, Cancer

## Abstract

In life-threatening conditions such as cancer and rare diseases, where there is no cure and no U.S. Food and Drug Administration (FDA)-approved therapy, patients sometimes seek access to an unapproved, experimental therapy through expanded access programs as their last, best hope for treatment to save their lives. Since the 1980s, the policies and the practice of expanded access have evolved, but a common challenge remains that there is no obligation, and often little incentive, for manufacturers to offer expanded access programs, especially for individual patients. In recent years, online campaigns seeking access to an experimental therapy have become more common, paralleling growth in and representing an intersection of social media, digital health, and patient advocacy.

Mackey and Schoenfeld have examined the evolution of expanded access policy, practice, and trends, as well as case studies of online campaigns to access experimental therapies, to arrive at several recommendations for the future of expanded access. This commentary puts their paper in context, examines their recommendations, and suggests further reforms.

Please see related article: https://bmcmedicine.biomedcentral.com/articles/10.1186/s12916-016-0568-8

## Background

Mackey and Schoenfeld examined the evolution of expanded access policy in the United States, as well as more recent trends toward alternative access such as “right-to-try” legislation and social media campaigns [1]. They discussed both the ethical and practical concerns behind manufacturer denials of expanded access [1].

There is no legal obligation for manufacturers to provide experimental therapies to patients prior to U.S. Food and Drug Administration (FDA) approval outside of a qualifying clinical trial [2]. In the absence of such an obligation, and in light of other perceived impediments to accessing promising therapies, patient advocates have sought alternative means of access via lawsuits, state and federal legislative and regulatory reforms, and publicity. Some reforms seek to loosen the restrictions involved in the FDA expanded access program (EAP) requirements, while others seek to obligate or pressure manufacturers to provide experimental therapies to patients individually or through an EAP. There is little correlation of factors that point to a “winning” strategy for obtaining such access [1]. Significantly, manufacturers, advocacy organizations, and the FDA each appear to be in search of a new paradigm that ethically and practically addresses patient and manufacturer concerns.

## Social media campaigns for expanded access: an intersection of trends

In an era where the average person spends over 6 hours per day online, including 1.72 hours on social media [3], we see the percentage of people who manage aspects of their health online continue to grow, from self-diagnosis via search engines to fitness apps to online access to medical records [4]. Similarly, the already growing trend of social media engagement has begun to intersect with digital health in areas such as social aspects of fitness tracker and dieting apps and even crowdsourcing diagnoses [5, 6]. Finally, a third trend, the rise of patient advocacy, has also paralleled and been influenced by the above trends in digital health and social media [7, 8].

Therefore, it should not surprise us when potentially life-saving recourse is also sought online via social media. Online campaigns seeking access to unapproved therapies offer hope for individuals and families facing a devastating diagnosis for which there is no alternative treatment.

**Fig. 1:**
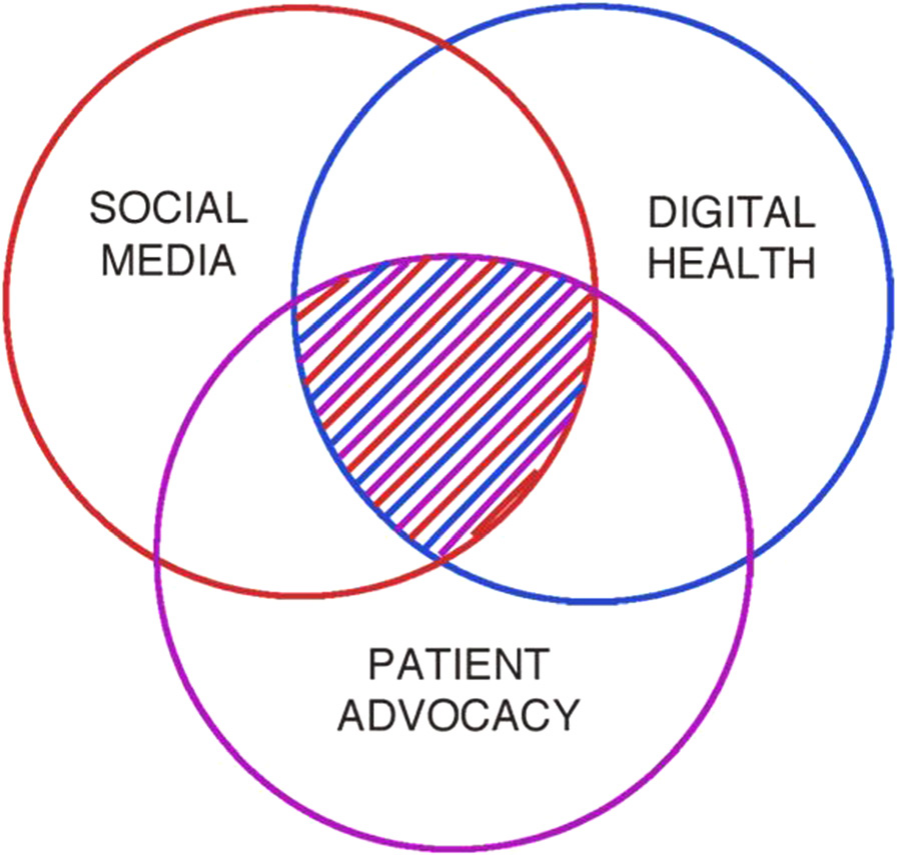
The intersection of trends in social media, digital health, and patient advocacy. The popularity of online social media campaigns to access experimental therapies operate in the shaded area where the trends of social media, digital health, and patient advocacy intersect. Absent a disruptive force, as these three trends continue, such campaigns will likely increase and grow more sophisticated in their implementation

Although a wide range of success and failure results from such campaigns, their proliferation results from the intersection and symbiotic relationship among trends in social media, digital health, and patient advocacy as shown in Fig. 1.

In addition, patient advocates have become increasingly vocal in campaigns for legislative or regulatory changes that purport to offer easier access to experimental therapies for patients and families facing life-threatening diagnoses, such as right-to-try bills and FDA regulatory reforms.

Against the backdrop of those individual and policy campaigns, pharmaceutical companies, regulatory agencies, and commentators have also begun attempting to shift the paradigm for accessing experimental therapies [9–11]. The question is whether those efforts, and others that may follow, are sufficiently disruptive forces that will alter the growth and sophistication of online campaigns for experimental therapies.

### Expanded access: evolution or revolution?

Mackey and Schoenfeld examined the ethical and practical questions posed by the evolving landscape of access to experimental therapies, identifying and analyzing the impact of a spectrum of social media campaigns, the right-to-try legislative movement, and the existing regulatory framework [1].

They accurately elucidate many of the pivotal points in the evolution of expanded access: 1) the issuance of FDA regulations regarding expanded access during the HIV/AIDS cases in the early 1980s; 2) the 2001 formation of the Abigail Alliance and its subsequent litigation; 3) the landmark 2008 decision of the U.S. Court of Appeals for the D.C. Circuit ruling that patients did not have the constitutional right to access experimental drugs; and 4) the evolution of FDA guidance on expanded access categories and processes from 2009 to 2013 and new revisions in 2015 [1]. I would add a most recent pivotal point involving one of the prominent social media campaign case studies, the 2014 Josh Hardy–Chimerix case. In that case, the very public promotion and resulting success of the campaign in accessing the experimental therapy (and the resulting positive outcome for the young patient, who recovered after receiving the drug) appeared to publicly validate social media as a promising strategy when alternatives have failed [9].

The Josh Hardy campaign illustrates that the most common hurdle to patient access is often not the FDA, which approves the vast majority (99 %) of expanded access requests it receives, most of which are for single-patient emergency or non-emergency use [12]. Instead, the critical challenge for most patients seeking expanded access is obtaining the approval of the manufacturer of the therapy. It is the refusal of manufacturers to provide access that becomes the subject of most patient and caregiver social media campaigns seeking expanded access.

The rationale offered by manufacturers for denying expanded access requests generally fall into one or more of the following: limited safety and efficacy data; limited supply of medication; the need to focus financial, personnel, and other resources toward clinical trials and drug approval; the potential impact on clinical trial enrollment; concern over potential poor outcomes and the effect upon the drug’s development of reporting of adverse events to FDA; as well as the ethical dilemma of expanding access to one or more patients versus an entire community [13].

### Patient case studies

Mackey and Schoenfeld identified 23 recent U.S. patient case studies who sought expanded access to experimental treatment and coded each for types of platforms used, use of multimedia, number of signatures obtained (for campaigns using online petitions), category of disease addressed, type of treatment requested, and name of company/organization petitioned [1].

Although their analysis was able to identify two common themes: 1) a common narrative among the campaigns of the denied drug representing their last and best hope for a life-saving intervention; and 2) the trend of higher petition signatures/social engagement correlating with greater national news attention, there was no correlation among factors supporting a particular “winning” strategy for obtaining access to the sought therapy [1].

### Implications for patients and policy

The Mackey and Schoenfeld study has potential implications both for patients as well as policy reforms. In part due to the success of the Hardy–Chimerix case and others, and absent a disruptive force, the trend of patient social media campaigns for expanded access will likely only increase. The strategies employed by the patient case studies will be replicated and improved upon by the next campaign and the one thereafter. The patchwork of proffered solutions by many parties might improve aspects of the expanded access system, such as patient education, patient–treatment match, and process streamline, but until a solution is adopted that reaches the core concerns of all parties and offers an alternative pathway, the status quo will remain steady.

Considering the framework of four consensus principles for expanded access reform proposed by Sanghavi et al. – Anticipation, Accessibility, Accountability, and Analysis [10], the Mackey and Schoenfeld study and proposed reforms fall squarely within those principles. However, those four suggestions should be joined by two additional “A” principles to shape a truly disruptive force regarding expanded access sufficient to constitute a valid alternative to social media campaigns or right-to-try legislation: Access and Advocacy.

#### Access

Mackey and Schoenfeld identified the common theme in the patient case studies that the sought treatment was viewed to be the patient’s last and best alternative (in most cases) to save their life [1]. Without alternative treatments, the only means by which a disruptive force can address this concern is to provide an actual pathway to drug access for appropriate patients. In a new expanded access paradigm, companies can better provide that pathway if they not only anticipate the requests, but have an obligation to provide access in appropriate circumstances. Not unlimited access, but a fair process at access.

The means, parties, and precise methods by which access would be created would certainly be the subject of debate; however, a broad continuum of both means – legislative, collective, or voluntary, and parties affected – the entire pharma industry, members of trade organizations, or individual companies, is possible. Proposed and implemented examples of this already exist in the notion of a national Expanded Access Institutional Review Board suggested by Caplan and Moch (Moch being the CEO of Chimerix during the Josh Hardy campaign) [9] and the independent Compassionate Use Advisory Committee being piloted by Johnson & Johnson, who interestingly, has not shied away from the term “compassionate use” as has become common in their industry [11]. Even considering potential legislative solutions, there is a continuum of method possibilities from requiring EAPs during pivotal trials under certain circumstances such as rapidly progressing childhood diseases or linked to designations such as the breakthrough designation, to individual Investigational New Drug (IND) applications evaluated by an independent commission. Even financial incentives to offer expanded access at various stages could be one end of a broader access spectrum. Given that the lack of treatment alternatives is what forms the basis for the social media expanded access campaigns, only a means of fairly providing sought therapies, at least to some patients, would constitute a disruptive force to the status quo.

#### Advocacy

Despite FDA assurances that expanded access uses have never prevented the approval of a drug [14], companies still fear the loss of millions of dollars invested in a drug development program, whether as a result of FDA scrutiny or public perception affecting their stock price, if a patient outside the controlled environment of a clinical trial suffers a negative or even neutral response. However, patients who wage sophisticated social media campaigns, and gain public support behind them, usually do so because there is meat on the bone – safety and efficacy data exist, at least in logical quantities, such that laymen see the sought treatment as a viable alternative … to death. Identifying the potentially appropriate cases for expanded access (e.g. patients meeting defined safety criteria), and what reforms might further assuage such fears, are a necessary predicate to the design of a disruptive force reform.

## Conclusions and call for a disruptive force

The intersection of trends in social media, digital health, and patient advocacy have created an environment where expanded access campaigns offer the last, best hope to save the lives of some patients. This trend will likely continue absent a disruptive force specifically addressing the previously published concerns of anticipation, accessibility, accountability, and analysis, but also access and advocacy, such that it creates a meaningful pathway for appropriate patients to access experimental treatments.

Mackey and Schoenfeld recommend policy reforms that respond to several of the concerns discussed above, specifically they suggest an Expanded Access Task Force, a centralized database of EAP policies and programs, a single point of EAP contact, published criteria for approval under an expanded access program, and anticipated response times. In addition, they suggest that economic incentives for implementing EAPs might be a valuable way to encourage them.

While the authors’ recommendations represent potentially worthwhile reforms, they should be viewed as pieces of a new expanded access paradigm intended to constitute a disruptive force in that space. Without evaluating proposed reforms of disparate parties against the core concerns of patients seeking access, and companies holding access, to investigational therapies, it is impossible to assess their true impact. Only a disruptive force offering an access pathway and advocacy that addresses industry concerns over potentially negative effects will alter the current trajectory of social media-based expanded access campaigns as the method of choice for desperate patients and caregivers.

## Abbreviations

EAP: expanded access program
FDA: U.S. Food and Drug Administration
IND: Investigational New Drug
